# Supplementary material for: Parasitic infection increases risk-taking in a social, intermediate host carnivore
Source: Commun Biol. 2022 Nov 24;5:1180. doi: 10.1038/s42003-022-04122-0 (PMC9691632; doi:10.1038/s42003-022-04122-0)
Supplement: Supplementary file 2 — Supplemental Information [file 42003_2022_4122_MOESM2_ESM.pdf]

variable 1	variable 2	type of correlation test	test statistic	p-value
**AIM 1 analysis**				
sex	coat color	Pearson's chi square	0.386	0.534
sex	social status	Pearson's chi square	1.230	0.267
sex	cougar overlap index	Pearson's chi square	0.484	0.785
sex	age in years	logistic regression	-0.690	0.491
age in years	coat color	logistic regression	0.520	0.605
age in years	social status	logistic regression	-1.401	0.081
age class	cougar overlap index	Pearson's chi square	11.450	0.075
coat color	social status	Pearson's chi square	0.050	0.824
coat color	cougar overlap index	Pearson's chi square	4.732	0.094
social status	cougar overlap index	Pearson's chi square	1.226	0.542
**AIM 2 analysis**				
sex	age class	Pearson's chi square	0.198	0.978
sex	system	Pearson's chi square	0.205	0.651
sex	time available	logistic regression	-1.140	0.253
time available	system	logistic regression	-1.070	0.286
system	age class	Pearson's chi square	1.786	0.618

Supplementary Table 1: Correlation tests between relevant variables for study Aims 1 and 2. Type of test changed based on the variable(s) being continuous (age in years and time available) or categorical (remaining variables).
