# Supplementary material for: Parasitic infection increases risk-taking in a social, intermediate host carnivore
Source: Commun Biol. 2022 Nov 24;5:1180. doi: 10.1038/s42003-022-04122-0 (PMC9691632; doi:10.1038/s42003-022-04122-0)
Supplement: Supplementary file 3 — Description of Additional Supplementary Data [file 42003_2022_4122_MOESM3_ESM.docx]

**Description of Additional Supplementary Files**

**File name:** Supplementary Data 1

**Description:** Source data behind figures in this paper
