# Supplementary material for: Parasitic infection increases risk-taking in a social, intermediate host carnivore
Source: Commun Biol. 2022 Nov 24;5:1180. doi: 10.1038/s42003-022-04122-0 (PMC9691632; doi:10.1038/s42003-022-04122-0)
Supplement: Supplementary file 5 — Reporting Summary-New [file 42003_2022_4122_MOESM5_ESM.pdf]

nature portfolio

Corresponding author(s): Connor J. Meyer

Last updated by author(s): Sep 24, 2022

Reporting Summary

Nature Portfolio wishes to improve the reproducibility of the work that we publish. This form provides structure for consistency and transparency in reporting. For further information on Nature Portfolio policies, see our Editorial Policies and the Editorial Policy Checklist.

Statistics

For all statistical analyses, confirm that the following items are present in the figure legend, table legend, main text, or Methods section.

n/a	Confirmed	
☐	☒	The exact sample size (*n*) for each experimental group/condition, given as a discrete number and unit of measurement
☐	☒	A statement on whether measurements were taken from distinct samples or whether the same sample was measured repeatedly
☐	☒	The statistical test(s) used AND whether they are one- or two-sided<br>*Only common tests should be described solely by name; describe more complex techniques in the Methods section.*
☐	☒	A description of all covariates tested
☐	☒	A description of any assumptions or corrections, such as tests of normality and adjustment for multiple comparisons
☐	☒	A full description of the statistical parameters including central tendency (e.g. means) or other basic estimates (e.g. regression coefficient) AND variation (e.g. standard deviation) or associated estimates of uncertainty (e.g. confidence intervals)
☐	☒	For null hypothesis testing, the test statistic (e.g. *F*, *t*, *r*) with confidence intervals, effect sizes, degrees of freedom and *P* value noted<br>*Give P values as exact values whenever suitable.*
☒	☐	For Bayesian analysis, information on the choice of priors and Markov chain Monte Carlo settings
☒	☐	For hierarchical and complex designs, identification of the appropriate level for tests and full reporting of outcomes
☒	☐	Estimates of effect sizes (e.g. Cohen's *d*, Pearson's *r*), indicating how they were calculated

Our web collection on statistics for biologists contains articles on many of the points above.

Software and code

Policy information about availability of computer code

Data collection	Microsoft Access and Excel were used to organize all data
Data analysis	Stata 13 was used for all analyses

For manuscripts utilizing custom algorithms or software that are central to the research but not yet described in published literature, software must be made available to editors and reviewers. We strongly encourage code deposition in a community repository (e.g. GitHub). See the Nature Portfolio guidelines for submitting code & software for further information.

Data

Policy information about availability of data

All manuscripts must include a data availability statement. This statement should provide the following information, where applicable:

- Accession codes, unique identifiers, or web links for publicly available datasets
- A description of any restrictions on data availability
- For clinical datasets or third party data, please ensure that the statement adheres to our policy

The datasets generated and analyzed during the current study are available from the corresponding author on request.

Field-specific reporting

Please select the one below that is the best fit for your research. If you are not sure, read the appropriate sections before making your selection.

☐ Life sciences ☐ Behavioural & social sciences ☒ Ecological, evolutionary & environmental sciences

For a reference copy of the document with all sections, see nature.com/documents/nr-reporting-summary-flat.pdf

Ecological, evolutionary & environmental sciences study design

All studies must disclose on these points even when the disclosure is negative.

Study description	We tested gray wolf blood serum, collected between 1995 and 2020 and cougar blood serum collected between 1999-2004 and 2016-2020 in Yellowstone National Park, Wyoming, USA, for exposure to the parasite Toxoplasmosis gondii. Samples were collected from 229 unique wolves and 62 unique cougars and, including some that were tested more than once, a total of 273 tests for seropositivity were done. We also collected wolf demographic data (age, sex, coat color) during capture, and determined leadership status during subsequent field observations. Behavioral data was collected over a wolf's entire monitored time (ranged from several weeks to over 12 years). We used a published cougar density map of Yellowstone to calculate a wolf's overlap with areas of high cougar density. We used this and the demographic factors to test which wolves are more likely to be seropositive. We also tested if any of four behaviors we defined as associated with higher risk were informed by T. gondii seropositivity.
Research sample	Research sample included gray wolves and cougars, of the Order Carnivora, living in Yellowstone National Park, Wyoming, USA. Wolves were being monitored following reintroduction to Yellowstone in 1995-6 and 12 to 15 were captured and sampled each year. Approximately 40% of the wolves sampled between 1995 and 2020 were tested for T. gondii. Cougars were caught, collared, and sampled (n = 62) during two phases of research. From 1999 - 2004, and then from 2016-2020. This research sample was based on the amount of blood serum available after other tests throughout that time were completed. We did not base our sampling on any demographic category. While >90% of the wolves in Yellowstone in the first several years were radio-collared (and sampled) this number dropped to approximately 25% since 2011 and no demographic was targeted or excluded during capture.
Sampling strategy	We did not predetermine sample size. We based our sampling strategy on having at least 60 of each of the main categories (T. gondii positive and negative; male or female; basic age divisions, black or gray coat color, leader or subordinate, and disperser and nondisperser). During model selection we included a random effect of individual wolf and results indicated sample size was sufficient in all but one analyses. We noted in the text which analysis had a sample size too small to make inferences, every other analysis included a much higher sample size.
Data collection	All of the authors either collected physical samples or behavioral data for many years. Physical samples included collecting blood during gray wolf or cougar capture and radio-collaring operations in Yellowstone National Park. Behavioral data was collected by the authors and technicians over 25 years and relied on direct observation. Generally observations were achieved from the ground using a spotting scope and recording behavior from 1 to 5 km away, so as to not affect wild behavior. Data was also collected using aerial tracking flights at least 40 times per year. Fixed wing aircraft included a pilot and observer recording observations and taking photos for detail.
Timing and spatial scale	Data (samples) were taken starting in 1995 in Yellowstone National Park from gray wolves during capture operations. Generally 12-15 wolves were captured and >80% of the time blood was taken during processing. If enough blood was collected for other tests, the remaining wolves were tested for T. gondii (approx 40% of all captured). The last wolves captured and tested (and included in this research) were sampled in December 2020. Cougars were caught and sampled from 1999 - 2004, and then from 2016 - 2020. All samples were taken within the boundaries of Yellowstone National Park.
Data exclusions	We chose to exclude the T. gondii test results from 1995-1999 in our tests for factors affecting seropositivity. Sampling indicates that the wolves did not have exposure (or enough exposure) to be infected until sometime around year 2000. In addition, wolves were translocated from Canada to Yellowstone in 1995 and 1996 and we wanted our research to focus on the carnivore system in Yellowstone, and avoid biases from individuals that had spent most of their life in other areas (northern Alberta, northern British Columbia, or northwest Montana) before translocation. In other published research from this same system, demographic studies usually begin a few years after reintroduction to avoid biases due to reintroduction.
Reproducibility	We did not have an experimental aspect to this research. If other researchers were able to sample individual (wild or domestic or laboratory) animals and observe their behavior, they would be able to use this research as a guide to reproduce the work in another species or system.
Randomization	Samples were not allocated into groups. One covariate split the sampled individuals into one of three categories based on their overlap with high cougar density areas in Yellowstone National Park. Individuals could have a 0 to 100% overlap with high density (based on their pack's minimum convex polygon each biological year). Many wolves had 5% overlap or less so we chose the divisions in order to maintain equal sample sizes in each category, low, moderate, and high overlap. Covariates were generally based on observations (sex based on external reproductive organs, age based on tooth wear, coat color based on color assessment). Behavior was either based on observation (leaders are the dominant wolves in the pack and scent-mark with the opposite sex leader), or post hoc based on overall movements summarized each 6 months (e.g. a disperser leaves the pack and does not return).
Blinding	Blinding was not relevant to this study.

Did the study involve field work? ☒ Yes ☐ No

Field work, collection and transport

Field conditions	Field conditions range from cold, long winters with temperatures reaching -20 degrees to summers reaching 75 degrees. Captures were done in the winter. Some observations used a fixed-wing aircraft flying less then 500 feet above ground.
Location	All field work was done in Yellowstone National Park and wolf observations and capture generally occurred between 2000 and 2500 meters in elevation.
Access & import/export	Most of the ground research was done in northern Yellowstone where vehicles, both public and park service, can access year-round. Fixed wing aircraft were permitted by the National Park Service Special Uses permit to fly at altitudes less than 500 feet for animal tracking. No import or export of samples was needed.
Disturbance	This study did not contribute to disturbance.

Reporting for specific materials, systems and methods

We require information from authors about some types of materials, experimental systems and methods used in many studies. Here, indicate whether each material, system or method listed is relevant to your study. If you are not sure if a list item applies to your research, read the appropriate section before selecting a response.

Materials & experimental systems

n/a	Involved in the study	
☒	☐	Antibodies
☒	☐	Eukaryotic cell lines
☒	☐	Palaeontology and archaeology
☐	☒	Animals and other organisms
☒	☐	Human research participants
☒	☐	Clinical data
☒	☐	Dual use research of concern

Methods

n/a	Involved in the study	
☒	☐	ChIP-seq
☒	☐	Flow cytometry
☒	☐	MRI-based neuroimaging

Animals and other organisms

Policy information about studies involving animals; ARRIVE guidelines recommended for reporting animal research

Laboratory animals	The study did not involve laboratory animals.
Wild animals	A total of 229 gray wolves (116 males, 112 females and one hermaphrodite, of ages between 8 months and 12 years) and 62 cougars (33 females, 29 males) were captured for this study. Wolves were radio-collared for future tracking and blood was drawn to test for exposure to a parasite. Wolves were captured using one of two methods: aerial net-gunning or helicopter-darting. Wolves were drugged (with a dart by helicopter, or by hand-injection with syringe if netted) with Telazol and took approximately 5 minutes to be fully sedated. After processing and metabolizing the capture drugs the wolves generally found their pack mates again within three to 12 hours. Cougars were caught and collared using cougar hounds released on fresh cougar tracks. Once the cougar was found, and had climbed a tree, the cougar was drugged (with a dart) with Ketamine and Metatomadite. It took ~10 minutes for the cougar to be fully sedated, at which point, project individuals would climb the tree and safely lower the cougar to the ground, where blood was drawn and the animal was outfitted with a radio-collar. These operation is part of a long-term National Park Service monitoring program and continued after this project. Animals generally wore the radio-collars (which enabled observers to determine their covariates for this study) until death or, occasionally, when collared were triggered to fall off remotely.
Field-collected samples	Blood serum samples (after centrifuging and dividing into plastic cryovials) were stored in a -80 degree freezer until being sent to Cornell Labs for the parasite serology tests. During shipping samples were sent in overnight packaging with dry ice.
Ethics oversight	All wildlife were handled in accordance with recommendations from the American Society of Mammalogists. Wolf capture protocols are approved by National Park Service veterinarians and University of Montana IACUC #AUP 046-21. Cougar capture protocols are approved by National Park Service veterinarians under IACUC numbers IMR_YELL_Stahler_cougar_2018.A1 and 1988-YCW-502.

Note that full information on the approval of the study protocol must also be provided in the manuscript.
